# Distributing foil from needle and syringe programmes (NSPs) to promote transitions from heroin injecting to chasing: An evaluation

**DOI:** 10.1186/1477-7517-5-24

**Published:** 2008-07-21

**Authors:** Rachael Pizzey, Neil Hunt

**Affiliations:** 1 Turning Point, 3rd floor Maltravers House, Petters Way, Yeovil, Somerset, BA20 1SH, UK; 2 KCA (UK); The Centre for Research on Drugs and Health Behaviour, London School of Hygiene and Tropical Medicine, c/o 55 Mackenders Lane, Eccles, Kent, ME20 7JA, UK

## Abstract

**Background:**

The report presents evaluation results from an intervention using specially produced foil packs to promote a transition from heroin injecting to inhalation (chasing) with injecting drug users (IDUs) attending four needle and syringe programmes (NSPs) in south west England.

**Methods:**

Service activity/uptake measures, brief structured interviews.

**Results:**

Out of 320 attenders, 54% took the foil packs when they became available. Over the period of the evaluation, NSP transactions increased by 32.5% from 1,672 to 2,216. Additionally, 32 new clients (non-injecting heroin users) started attending the service to obtain the foil packs. This group would not otherwise have been in contact with the treatment service. More detailed data from one site are reported for 48 recent injectors who took foil within the NSP where the piloting first commenced. Prior to the introduction of the foil packs, 46% of this sub-group reported chasing heroin in the previous four weeks. At follow up, 85% reported using the foil to chase heroin on occasions when they would otherwise have injected. Among the people who took it, client satisfaction with the quality and size of the foil packs was good and respondents viewed its availability as a valuable extension to the NSP's services.

**Conclusion:**

These findings suggest that distributing foil packs can be a useful means of engaging NSP attenders in discussions about ways of reducing injecting risks and can reduce injecting in settings where there is a pre-existing culture of heroin chasing. Further research should see whether these findings can be reproduced in other cultural contexts and evaluate whether the observed behavioural changes are sustained and lead to reductions in harm including blood-borne infections and overdose.

## Introduction

Injecting is the most hazardous way of using heroin. One approach to reducing drug-related harm among people who are unable or unwilling to stop using heroin involves 'route transition interventions' that aim to shift episodes of drug administration from injecting towards methods that are less risk-laden [1]. This report discusses the development of packs of aluminium foil intended for heroin smoking and distribution within needle and syringe programmes (NSPs) as a tool to aid interventions that promote a switch from injecting to heroin smoking (chasing). It provides an account of the initial experience of distributing the foil as part of an intervention, its acceptability and impact within four English NSPs.

## The risks of injecting versus smoking heroin

When compared to other routes of heroin administration, injecting introduces many specific risks and simultaneously increases others. The risk of acquiring blood-borne viral infections including HIV, hepatitis B and hepatitis C are all strongly associated with the sharing of injecting equipment through blood to blood transmission. Poor injecting hygiene can also lead to a large number of local and systemic bacterial and fungal infections [2]. Injecting also causes soft tissue injuries and often leads to long-term physical damage such as collapsed veins as a result of the repeated physical trauma that arises from repeated injecting [3]. When compared to inhaling/smoking heroin, the risk of overdose is far greater when people inject [4]. Additionally, there is suggestive evidence that severity of dependence is higher among people who inject; however, the direction of causality here is less clear, as severity of dependence may also lead to injecting [5].

Heroin smoking is not without its own harms. Dependence certainly occurs and (long term) heroin smoking is associated with respiratory health problems, although research has not yet adequately quantified these risks or distinguished them from confounding factors that are common among heroin users such as tobacco and cannabis smoking. Although the risk of overdose is lessened when heroin is inhaled, it is not eliminated [4,6]. There are also case reports of Progressive Spongiform Leukoencephalopathy that have been associated with heroin smoking [7,8]. There can therefore be no suggestion that smoking heroin is without harm. Nevertheless, on balance, there is good reason to suppose that among people who would otherwise inject and are not ready to stop their heroin use, making a transition to heroin smoking would be a beneficial step.

## The potential for transitions interventions within a harm reduction model

Research has shown that transitions both towards injecting and away from it can occur spontaneously and without intervention [9-11]. This observation helped provide a foundation for developing interventions that aimed to affect this process more deliberately. Injecting can be reduced either by a) decreasing the number of people who commence injecting or b) increasing the rate at which people stop injecting (either completely or partially) i.e. a 'reverse transition' away from injecting. Oral opioid substitution treatment is the most widely used intervention of this sort, as it operates by reducing the injection of street opiates and replacing this, to a greater or lesser extent, with oral drug administration. Because of the particular risk of injecting, any injection event that is avoided may reduce risk, even if the drugs are still used by some other means. A number of possible opportunities for intervention have been discussed including campaigns that promote reverse transitions to heroin chasing to IDUs [1]. Within the UK, where heroin is almost exclusively smoked or injected, social marketing campaigns and harm reduction materials have previously been developed that aim to encourage transitions to chasing among injectors such as the Healthy Options Team (HOT), East London's Chasing Campaign [12] and Lifeline, Manchester's 'Smoking Brown' leaflet [13]; however, there are no published evaluations of these, or of services that have provided foil directly as part of an intervention to encourage a reverse transition from injecting to smoking.

## Development of the foil packs

Exchange Supplies (ES) is a social enterprise that has previously been involved in developing and promoting route transitions interventions including the 'Break the cycle' campaign to prevent initiation into injecting based on work by Hunt and colleagues [14]. To complement this, ES has developed packs of aluminium foil designed for heroin chasing that can be used to support interventions to promote reverse transitions within NSPs. During the development of the materials a small reference group of heroin users were consulted to try to ensure that the foil would be acceptable for heroin smokers. The packs contain 50 sheets and use a high quality foil that is 18 microns thick and in sheets sized 200 mm by 125 mm, which broadly corresponds with the size that people commonly reported using as a 'tray' for running the heroin. Kitchen foil thickness varies with manufacturers but cheaper commercial foil may be as thin as 10–11 microns.

Non-toxic vegetable oil is sometimes used as a lubricant during the manufacture of Aluminium foil. UK heroin smokers often run a flame over the foil before use to burn this oil off, and as it does so there is a small amount of visible smoke. There is anecdotal evidence that some heroin smokers are anxious about the content of this coating, and believe it to pose some risk to their health. The ES foil is manufactured without any such coating as, although there is no evidence of health risk from the oil coating, an absence of oil is a feature that heroin smokers regard as desirable.

Each pack of foil contains information that explains the purpose of the materials "to encourage people who inject heroin to smoke it instead and encourage those who smoke heroin to make contact with treatment services." Information on the risks of heroin smoking is included along with a link to a campaign website [15].

Access to the website is password protected with the password 'HarmReduction' to restrict access to the information for the general public. The site contains: detailed 'practice notes' for drug workers; case studies; and, pipe-making guidance to assist people who are unfamiliar with smoking technique.

## Legal considerations

Within the UK, the knowing supply of articles for the administration of controlled drugs is prohibited under section 9a of the Misuse of Drugs Act (1971). Recognising the role of harm reduction services, a legal exemption has been introduced to permit the supply of needles and syringes within NSPs. Subsequent amendments have also been made with the express intention of permitting the provision of other equipment that can reduce drug related harm, such as citric acid, water for injections, and filters. Foil has not yet been added to the list of exempt items and therefore its supply to drug users is, technically, forbidden under section 9a. However, there is considerable experience of this situation within the UK, as the law has often lagged behind harm reduction practice. UK services have been providing items to reduce harm in contravention of Section 9a of the Misuse of Drugs Act (MDA) for many years, and changes to bring provision within the law have tended to follow practice. No service has ever been charged with this offence.

Under British law there is a separation between investigation and prosecution. An independent body – the Crown Prosecution Service (CPS) – is responsible for placing criminal charges before the court, but it can only do so on the basis of evidence provided by the Police, and it cannot instigate investigations. A test which the CPS apply to any file with which they are presented is the 'public interest test' i.e. would the successful prosecution of this case be in the public interest. The fact that the items are being supplied to prevent harm would mean that even in the absence of an agreement with the police, a prosecution would be unlikely to pass this test [16].

In order to be absolutely certain, the service – Turning Point – liaised with local police, who were supportive of this initiative. The police provided a letter of support, which made it clear that they would not consider provision of foil to be an offence that should be prosecuted, and advised Turning Point that they had no plans to prepare a file for the CPS. This meant that the foil could be provided in a way that minimised the risk of prosecution.

## The research questions

The main research questions were: Will injecting drug users (IDUs) attending NSPs take foil intended for heroin chasing if it is offered by services? Does foil distribution encourage attendance by non-injecting heroin users not in contact with the service?

Does foil distribution increase the number of face to face contacts within NSPs? Does foil distribution produce behaviour changes away from injecting? Are the foil packs regarded as acceptable for heroin chasing by heroin users?

## Methods

The study was undertaken between October 2006 and August 2007. The evaluation took place in NSPs run by a national drugs charity – *Turning Point *– within four small/medium sized towns in South West England. The services employ specialist harm reduction practitioners whose role is to work with injecting drug users. During the evaluation, these personnel offered the foil to people attending the service with an explanation of the intervention objectives and a discussion about the relative risks of injecting and chasing. They also collected the evaluation data.

The service's basic information system routinely gathers a short set of data from attenders. Basic descriptive data include: gender, ethnicity, age and preferred drug, however, the service deliberately avoids requiring too much information in order not to undermine its work and deter the target population. A simple amendment was made to the existing information system to monitor whether attenders had been offered foil and enable follow-up questions to be asked. Additionally, a short (one A4 page) questionnaire was developed for administration by the workers as a structured interview. This was only used within one service where the first author was based. The questionnaire responses were collected by five practitioners who worked within the NSP. Staff were briefed about the aim of the programme and data gathering requirements but did not require extensive training to administer the questionnaire as this only required a modest extension to their existing data gathering role and addressed topics which they were experienced at discussing. The questionnaire was used when someone took foil for the first time and incorporated a short set of questions about: whether people had ever previously chased heroin (to clarify whether would need training in the skills needed to chase heroin); whether people had chased heroin in the previous four weeks (i.e. whether they were current/recent chasers); and, whether they wanted to try the foil. Follow up data about use of the foil were subsequently collected along with a short, note-based qualitative record of clients' comments about the suitability of the foil and whether or how they had used it. Follow up data were usually gathered at the person's next visit to the service. The interval between giving out the foil and follow up was variable, reflecting the patterns in the way people attend NSPs, but generally occurred with four weeks. Because the work took place outside of the National Health Service and the additional data were anonymised, NHS research ethics approval was not required. However, all records were subject to the same policies that are used for the service's clinical records with regard to matters such as client confidentiality and storage of records.

## Findings

Between October 2006 and August 2007 the NSPs were attended by 320 opiate users who made a total of 1672 visits. During this time, 174 people (54%) of those who attended the four services during the pilot chose to take the foil.

Foil was not offered to everyone as for some people there were other clinical priorities, which meant that there was not time to discuss the foil intervention. Furthermore, NSP staff particularly targeted femoral injectors and those people who indicated that they had problems with their injecting, as their need was judged to be greater, and the foil distribution provided a novel way to engage people in discussions about risks and how these might be reduced. Consequently, the foil uptake rate is best regarded as the minimum that could potentially have been achieved with this population (see Table 1).

**Table 1:** Uptake of foil distribution

NSP (population)	Date pilot commenced	N people attending from commence of pilot to August 2007	N (%) who took foil	N new heroin chasers attending services
Site 1 (42,000)	Oct-06	161	85 (53)	9
Site 2 (9,000)	Apr-07	45	23 (51)	9
Site 3 (58,000)	May-07	69	38 (55)	10
Site 4 (37,000)	May-07	45	28 (62)	4

TOTAL		320	174(54)	32

The mean age of the people who took foil was 32.5 years (range 18–59). Overall, women comprised 16.8% of the total population attending services; however, they were significantly more likely to take the foil (62.3%) compared to men (44.6%) (Chi squared 6.408, df 1, p = 0.016).

It was not possible to obtain feedback from everyone who took foil as NSP attendance is variable and some people did not re-contact the service during the period. Furthermore, the nature of the NSP service is 'low threshold' and practitioners had to balance the desire to get feedback with the need not to deter people by being over-intrusive and, attending to the clients' own concerns, which were always given priority.

When compared with the equivalent period of time, the overall number of visits to the services increased by 32.5% after the foil packs were introduced (see Table 2), however, the increase in visits did not occur evenly across services, ranging from 12.8% to 79.3%.

**Table 2:** Visits to the services before and after the introduction of the foil packs

	Pre-pilot period	Pilot period	Increase in visits
NSP 1	1105	1433	29.7%
NSP 2	187	211	12.8%
NSP 3	196	242	23.5%
NSP 4	184	330	79.3%
Total	1672	2216	32.5%

It is also of note that, during the study, 32 new people attended the service who chased heroin but did not inject.

In the initial pilot site, where the first author was based, the additional questionnaire data were sought from all people who took the foil. This generated an eventual sample of 48 people. Of these, 11 (22.9%) were women. Mean age was 32.4 (range 20– 59). The sample included intermittent injectors who only injected every three to four weeks through to people injecting 10 times daily. Everyone had previously smoked heroin and was therefore familiar with this technique. Within the four weeks before being given the foil, 21 (46%) of the sample had smoked heroin on one or more occasions (data were missing for two cases), indicating that a substantial proportion of the sample routinely mixed chasing and injecting rather than using one method exclusively.

At follow up, all but two people reported using the foil and 41 (85%) reported chasing on one or more occasions when they would otherwise have injected. This included 22 people who previously reported that they had not smoked heroin in the four weeks prior to receiving the foil. The frequency with which the foil was used varied. Some people only used it once or twice experimentally and then reported that they were unlikely to use it again, others reported marked changes in their pattern of use with chasing supplanting injecting on all or almost occasions; however, among the foil users most people reported that having the foil available led to a partial replacement of injecting with chasing. In response to the question "Do you think having foil available in the needle exchange is helpful?" all 48 respondents agreed.

The foil provision and accompanying discussion with the practitioner often seemed to act as a trigger to review injecting that coincided with accumulating concerns about risk and harm e.g. difficulty finding veins. Several people reported that because the foil was provided free, they had it available when it otherwise would not have been due to a lack of money, because of the inconvenience of buying it, or because of embarrassment about buying foil due to shopkeepers assuming or knowing that it was for heroin use. People also reported having been refused foil by shop assistants for this reason. One man explained that he always smoked rather than injecting after drinking alcohol as an overdose prevention strategy. It is not certain that this could be directly attributed to the foil distribution, but this points to an overdose prevention message that might usefully be emphasised as part of the intervention.

A range of negative factors associated with chasing were reported including: the lower intensity of the experience; experiencing nausea that did not occur when injecting; getting dirty fingers from the soot that accumulates underneath the foil; and, the smell of the heroin, which some people found unpleasant and which also made it more difficult to conceal use of the heroin.

Among people who initially said that they did not want to try the foil, many then went on to use it at a later date, either when they started treatment and particularly wanted to avoid injecting or, as their injecting became more problematic. People often used it intermittently, stopping and starting several times over a period of months, especially when their venous access became poor. One woman started to routinely split her heroin in two so that she could smoke half if she was unable to find a vein easily. In other cases, people used it to wean themselves off injecting when they started receiving oral substitution treatment or to manage a lapse after detoxification.

There appeared to be some beneficial social network effects as several IDUs took foil home for their smoking partners, some of whom were among the non-injecting heroin users who subsequently attended the service. Providing foil was also reported to have encouraged several people attending the service to persuade their partners to avoid or reduce injecting. Lots of people commented on the greater sociability of chasing heroin and sometimes smoked their heroin for this reason. In this regard, it is of note that this greater sociability was also a positive factor emphasised in the Healthy Option Team's original social marketing campaign to promote chasing to injectors. In one case, a low-level dealer took six packs at a time in order to promote smoking among his injecting customers.

Without exception, all respondents felt that foil provision was a useful adjunct to the services already offered within the NSP. Client feedback also suggested that the foil was often discussed with other people within their drug using networks and that the foil was also passed on to both injectors and chasers who did not attend drug services. As a result, 32 people who had never attended services before came to the NSP to obtain supplies of foil. In one instance, a man who had stopped attending the NSP re-engaged to obtain foil after an interlude of six years.

The majority of comments on the foil packs themselves were favourable. People appreciated the thickness of the foil and contrasted this with shop-bought foil which was thinner and sometimes developed holes when used. Most people felt the size of the sheets was about right, with a minority suggesting it should be bigger.

## Discussion

This study suggests that foil provision was regarded as worthwhile by a substantial proportion of the NSP attenders in the four towns in which the evaluation took place. Everyone who took the foil and provided feedback felt that the foil intervention should be provided in NSPs. This included people who did not go on to use the foil regularly themselves but nevertheless thought it was beneficial for others. The number of people who took foil and subsequently reported using it at times when they would otherwise inject provides further evidence of its potential utility and, the case of someone who re-engaged with the service after a break of six years also supports the view that foil provision is valued.

Regarding the acceptability of the foil, the majority of people were positive about it and felt it was well suited to its purpose. In particular, its thickness was appreciated as this contrasted favourably with the kitchen foil with which they were more familiar. For most people the foil size was just right though a small number of participants felt the sheets should be larger. Overall, there was no indication that its specification should be changed.

The finding that some heroin chasers visited the service for the first time in order to obtain the foil packs indicates potential for using this intervention to engage heroin users who are not injecting. This may provide opportunities for earlier intervention to reinforce their non-injecting status and deter them from beginning to inject, as well as encouraging engagement in the wider range of treatments that can either reduce reliance on street heroin or, ultimately, assist people to attain abstinence. One possible concern might be that non-injectors attending an NSP would be vulnerable to beginning injecting and that NSP attendance would facilitate this by increasing links with injectors. In the UK, some degree of mixing of non-injectors and injectors commonly occurs in other services such as opioid substitute prescribing or day programmes and among the non-injectors who came for foil it seemed that they were routinely exposed to injecting within their existing social networks. It would clearly be prudent for services to be attentive to this risk; however, this concern probably ought not be over-exaggerated, as the packs are explicit about the increased risks of injecting and ordinary kitchen foil is, in any case, a widely available product.

In each NSP the number of visits to the service increased markedly (between 13–79%) when foil was made available. Within the services there were no other initiatives over the same period that would obviously explain this. It is possible that this change was attributable to increases in the population of IDUs in each area, however there is no local evidence that supports this. Service users' favourable comments about the new service and the fact that some heroin chasers registered with the service to obtain foil strongly suggest that its distribution was responsible for at least some of this increase. It is, nevertheless, unclear whether any such effect would be sustained and this requires longer term follow up.

The greater propensity of women to take the foil and the possibility that foil distribution may particularly appeal to female IDUs is of note. Whether this can be explained by a greater readiness to adopt new interventions, lower levels of dependence or attachment to injecting, experiencing more problems with injecting or other factors is unclear and warrants further investigation.

Reports that people readily passed on foil packs and discussed the intervention with other existing heroin users point to some potential to exert beneficial effects beyond those drug users who are seen within services. When developing the intervention, consideration was given to the risk of promoting heroin use among people who had not used it before. To address this the foil is only distributed to known heroin users via NSPs and the part of the website that might be considered to contain more enabling information i.e. detailing pipe construction, is password protected. Among participants, there was no evidence that the provision of foil led people to promote heroin use among people who did not already use it; however, this risk merits more careful evaluation in a larger study.

These early experiences raise questions about the possibility of foil distribution contributing to broader cultural changes away from injecting. Recently, countries such as the Netherlands and Spain have seen marked shifts away from injecting to chasing [17,18]. Such trends are certain to be the product of multiple factors including the purity of the drug (i.e. whether it is readily and affordably smoked) and perceptions of serious epidemic illness associated with injecting – HIV/AIDS. It would be over-optimistic to assume that such a campaign could ever be a sufficient factor to deter injecting. Nevertheless, a sustained and focused intervention programme to promote transitions from injecting may help encourage or accelerate such trends where other conditions are favourable.

Within the towns in which the evaluation took place, it seemed that heroin injectors had almost invariably chased heroin previously. In this situation, the intervention aims are to promote reversion to a practice that people are already skilled in, through the provision of enabling materials, information and persuasion. We anticipated that some people would have been initiated directly into injecting and have no experience of chasing. In this scenario, people would also need to be taught the skills required to chase heroin, using materials such as those Lifeline's 'Smoking Brown' leaflet [13]. As efficient heroin chasing is a skill that is not always readily acquired, the intervention may not work so readily if dependent heroin users have to have a longer period of trial and error involving a degree of waste, and using technique that delivers a less intense experience. Subsequent research should be attentive to whether participants already possess the necessary skills to chase heroin, or need to learn them as part of the intervention, as this seems likely to have a bearing on its efficacy.

The study focused on heroin injectors and paid little attention to crack cocaine use. In the UK, crack is usually piped or, increasingly, injected with heroin as a 'speedball' [19]. It is unusual for British crack cocaine users to chase crack cocaine but there may be potential to adapt the intervention in this way. Future work could usefully examine whether it is feasible to use foil packs as the basis for an intervention to reduce risks from the use of crack cocaine.

This study has a number of identifiable limitations. The sample size is small and, although it includes several different towns, it is unclear to what extent the findings would be generalisable to other settings with differing local drug cultures. This may particularly apply to larger urban/metropolitan areas and would have particular relevance in settings where people start using heroin by injection rather than chasing it earlier in their drug using career. Nevertheless, the study indicates the feasibility of delivering this intervention within NSPs that are typical of many services within the UK.

The findings are largely limited to a short series of simple questions and client feedback gathered opportunistically by the practitioners who delivered the intervention during the usual operation of the NSPs – generally at the subsequent visits of those people who took the foil. Although the early findings are encouraging, they give no indication of whether the reported behaviour changes would be sustained, which would require longer term follow up. As with any self-reported behaviour, we cannot be certain that this provides an entirely accurate account of what happened, as it is subject to recall effects and may also be affected by social desirability responding. The objectives of the intervention were explicit and participants would have a clear sense of what the practitioners were trying to achieve. Having said this, many participants were well known users of the service with whom practitioners had a well established relationship. Central to this relationship is an ethos of acknowledging the risk-taking that is inherent to heroin use and talking about risk behaviours honestly and candidly. This may have helped limit any social desirability responding and the occurrence of a small number of participants who took the foil and subsequently reported that it did not suit them seems indicative of authenticity within the feedback.

Finally, the intervention was delivered by experienced NSP practitioners who had considerable enthusiasm for the project. It is uncertain how well these results would be reproduced in services where there is a lower level of commitment to the intervention or which employed less experienced staff.

## Conclusion

This study has confirmed the feasibility of providing foil as part of an intervention to promote a 'reverse transition' from injecting to chasing heroin within English NSPs. Reports from participants suggest that the foil packs are well suited to their purpose and that the intervention is seen as relevant by service users. Self reported behaviour change was in the desired direction, suggesting that providing foil within NSPs along with discussion about the benefits of chasing can help reduce risk-taking. Evidence that use of foil packs can help attract or re-engage heroin users with services is also encouraging. The study has a number of limitations, which make it desirable to replicate the findings and examine whether the short term impacts that were found are sustained or can be further developed. Although there was no evidence that distributing the foil led to adverse effects, this should be examined more systematically in any fuller evaluation.

## Competing interests

The evaluation was unfunded and RP received no financial support for the work, which was undertaken as an extension to her general duties. However, ES provided the foil packs to her service at no cost. During the past five years, NH has undertaken several paid consultancies from ES. Among them, he helped develop web-based materials for the foil packs and was commissioned to collaborate with the lead author to write up the findings from this evaluation.

## Authors' contributions

RP conceived the study, had full responsibility for its operational management and data collection and collaborated equally with drafting the paper, NH managed the data analysis and collaborated equally with drafting the paper.
